# Paediatric Mediastinal Lipoblastoma

**DOI:** 10.5334/jbsr.3166

**Published:** 2023-07-17

**Authors:** Vera Brazão de Carvalho, João Lopes Dias, Ana Nunes

**Affiliations:** 1 Centro Hospitalar Universitário Lisboa Central, PT

**Keywords:** Lipoblastoma, Lipomatous tumor, Mediastinum, Children, Hyperechoic mass, Fat suppressed mass

## Abstract

**Teaching Point:** Lipoblastoma should be considered in the differential diagnosis of painful rapidly growing fatty mass within the mediastinum in infants or young children under three years old.

## Case History

A 2-year-old boy presented with a nine-month history of progressively growing cervicothoracic painful mass, without associated inflammatory signs, respiratory distress, or constitutional symptoms.

On physical examination, he was haemodynamically stable, with normal oxygen saturation. Cardiopulmonary auscultation revealed a slightly decreased vesicular murmur in the upper left hemithorax. A non-adherent mass of elastic consistency was palpable in the left supraclavicular fossa. Laboratory findings were unremarkable.

**Figure 1:**
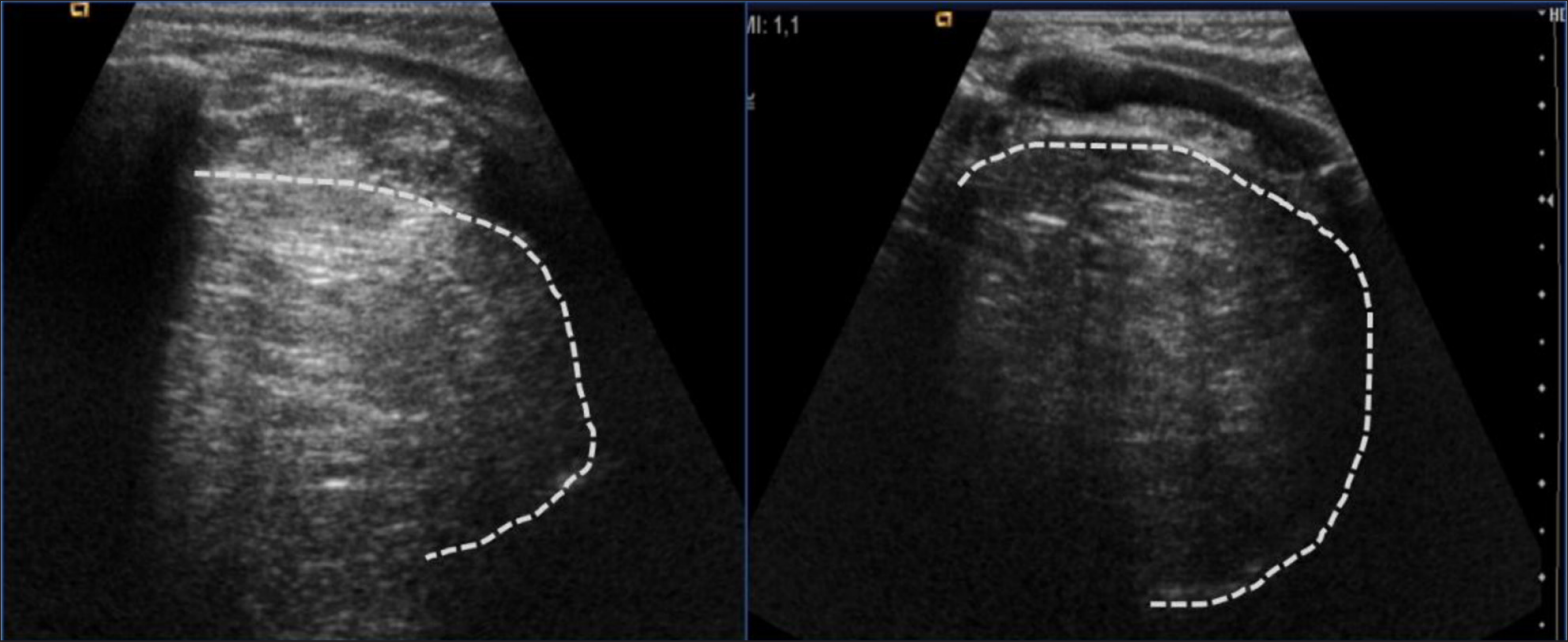


An ultrasound was performed, which showed a bulky hyperechoic and only slightly heterogeneous solid mass occupying the upper left hemithorax without calcifications or Doppler signal (Figure 1).

**Figure 2:**
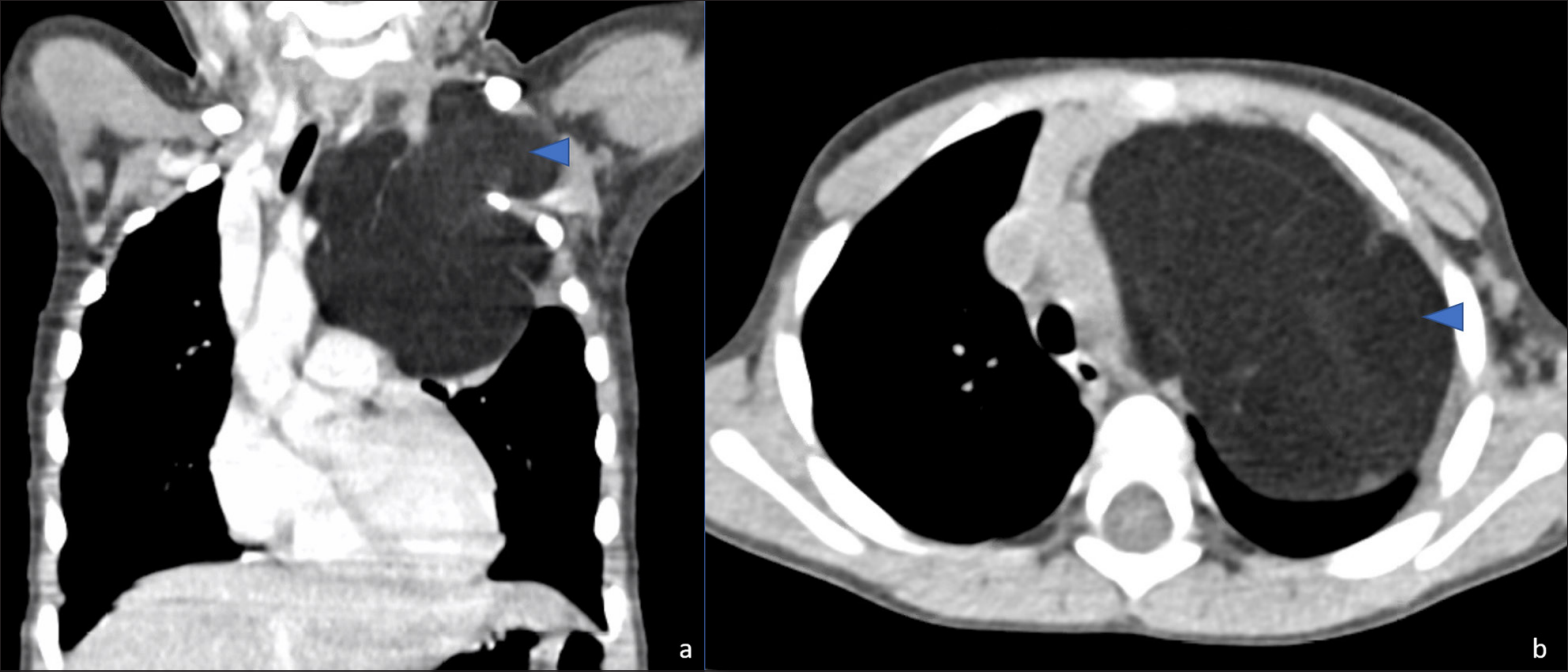


For better characterization, he underwent a chest computed tomography (CT) (Figure 2) that depicted well defined, expansive mass located in the superior mediastinum, extending into the left supraclavicular fossa, with a prominent fat density (ranging from –100 to -80 HU) and only a few thin and hypovascular septa (arrowhead), compatible with a lipomatous tumour. Compression of lung parenchyma and contralateral shift of superior mediastinal structures are seen.

**Figure 3:**
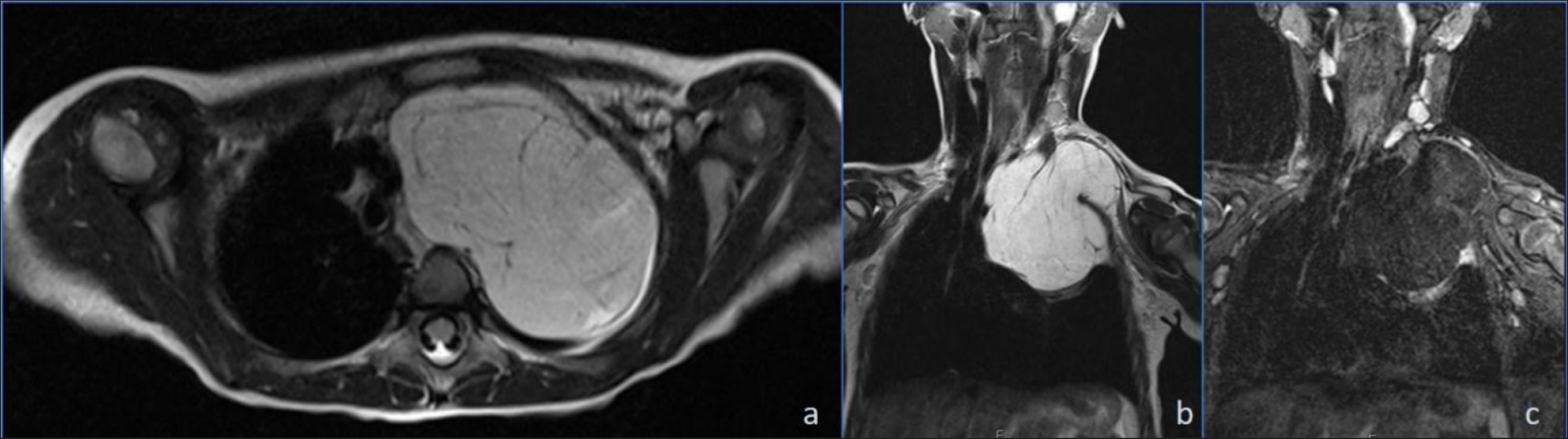


A complementary magnetic resonance imaging (MRI) was performed to precisely determine tumour margins, namely its deep extension. In Figure 3 a diffusely T2-hyperintense lesion is seen in axial (a) and coronal (b) planes showing signal loss in fat-saturated sequences (c), which is consistent with a lipomatous tumour. A similar behaviour was also seen in T1 WI (not shown). MRI high-contrast resolution was useful to assess the margins of the lesion. Although reaching the posterior mediastinum, it did not demonstrate transforaminal intraspinal extension or signs of local aggressivity.

The patient underwent a thoracotomy and complete free margin resection of the tumour. Histopathological results showed mainly adipocytes and lipoblasts at varying stages of maturation consistent with mediastinal lipoblastoma.

Two years after the procedure, no signs of recurrence were found.

## Comments

Lipoblastoma is a rare benign mesenchymal tumour of embryonal fat that occurs almost exclusively in less than 3-year-old infants. Almost half of cases appear in the limbs, followed by the trunk and head and neck. Mediastinum is an uncommon location [1].

Symptoms are mostly from local mass effect. When located in the mediastinum, it can cause dyspnoea, mainly due to lung and airway compression [1].

Cross-sectional imaging (CT or MRI) is very useful for establishing the lipomatous nature of the mass, determining its exact location and local extension which enables proper surgical planning. However, preoperative differential diagnosis between other fatty tumours is quite difficult [1].

Liposarcoma (particularly the myxoid variant) and hibernoma should be considered in the differential diagnosis, but both usually occur in older patients. Germ cell tumours, like teratoma, are frequent in the anterior mediastinum and more prone to demonstrate calcification and/or cystic component [1].

The definitive diagnosis is only achievable by histological examination [1]. Complete tumour resection should be the first choice in treatment of this condition [1].
